# Increased Levels of sRAGE in Diabetic CKD-G5D Patients: A Potential Protective Mechanism against AGE-Related Upregulation of Fibroblast Growth Factor 23 and Inflammation

**DOI:** 10.1155/2017/9845175

**Published:** 2017-09-25

**Authors:** Elena Dozio, Valentina Corradi, Elena Vianello, Elisa Scalzotto, Massimo de Cal, Massimiliano Marco Corsi Romanelli, Claudio Ronco

**Affiliations:** ^1^Department of Biomedical Sciences for Health, Università degli Studi di Milano, Via L. Mangiagalli 31, 20133 Milan, Italy; ^2^Laboratory of Molecular Pathology, I.R.C.C.S. Policlinico San Donato, Via R. Morandi 30, San Donato Milanese, 20097 Milan, Italy; ^3^Department of Nephrology, Dialysis and Transplantation, San Bortolo Hospital, Viale Rodolfi 37, 36100 Vicenza, Italy; ^4^International Renal Research Institute Vicenza (IRRIV), San Bortolo Hospital, Viale Rodolfi 37, 36100 Vicenza, Italy; ^5^Service of Laboratory Medicine 1-Clinical Pathology, I.R.C.C.S. Policlinico San Donato, Via R. Morandi 30, San Donato Milanese, 20097 Milan, Italy

## Abstract

Advanced glycation end products (AGEs) may induce cardiac remodeling in kidney disease by promoting fibroblast growth factor 23 (FGF-23) expression. Since AGEs are increased in diabetes mellitus (DM), our first aim was to evaluate the existence of any potential association between AGEs, FGF-23, inflammation, and increased cardiovascular risk in DM patients on dialysis (CKD-G5D). Secondarily, we explored the potential role of the soluble receptor for AGEs (sRAGE) as a marker of heart failure. Levels of glycated albumin (GA), sRAGE, c-terminal FGF-23 (cFGF-23), brain natriuretic peptide (BNP), and inflammatory mediators were compared between DM and non-DM CKD-G5D patients. The levels of sRAGE, cFGF-23, BNP, and proinflammatory markers were over the ranges of normality in both DM and non-DM groups. Only GA and sRAGE levels were increased in DM compared to non-DM patients. Plasma levels of sRAGE and CRP were the only independent predictors of BNP concentration. In conclusion, in DM CKD-G5D patients, sRAGE appeared to be a marker of cardiac remodeling. Indeed, its increase could be a potential protective mechanism against the increased risk of cardiovascular complications related to AGEs and inflammation. The causal relationship between sRAGE and cardiovascular risk in these patients needs to be further confirmed by mechanistic studies.

## 1. Introduction

End-stage renal disease (ESRD) in patients with chronic kidney disease (CKD) is a condition characterized by including volume overload, hyperkalemia, metabolic acidosis, hypertension, anemia, and mineral and bone disorders (MBDs), and it is considered a clinical model of premature aging. ESRD patients have an increased risk of different diseases, mainly at cardiovascular and cerebrovascular level, and have a mortality rate at least 20–30 times higher than what their healthy age-matched patients have [1]. ESRD is stricly related to cardiovascular diseaes (CVDs) through several mechanisms, which include the inflammatory response, the production of reactive oxygen species, the phosphate toxicity, and the activation of different endocrine pathways, such as the fibroblast growth factor 23 system (FGF-23) [1]. Also, MBDs represent a severe complication and an important mortality risk factor in CKD patients on dialysis (CKD-G5D) [2].

FGF-23 is a 32 kDa glycoprotein secreted by osteocytes which has been receiving great interest as a new risk factor for CVDs and death both in individuals with CKD [3, 4] and in adults with preserved kidney function [5, 6]. In particular, increased FGF-23 levels have been associated with vascular dysfunction, left ventricular hypertrophy, and the risk of heart failure, stroke, and death [3, 7, 8]. In CKD, FGF-23 levels increase as a compensatory mechanism to keep normal phosphate levels by inhibiting renal phosphate reabsorption and 1-alpha-hydroxilase activity, the key enzyme for calcitriol production [9]. Anyway, although this increase is acknowledged as a physiological protective mechanism, it could directly contribute to the onset and progression of inflammation and CVDs [7, 8, 10].

It has been recently observed that FGF-23 expression may be promoted *in vitro* by advanced glycation end products (AGEs) through the upregulation of NF-*κ*B [11]. Indeed, in a mouse model of renal failure, the activation of the cell-surface receptor for AGEs (RAGE) induced FGF-23 expression in cardiac fibroblasts and promoted cardiac remodeling [12].

Metabolic disorders including diabetes mellitus (DM) are characterized by high levels of AGEs that are key mediators of DM-related complications, inflammation, and aging. These products, generated by nonenzymatic reactions between reducing sugars and protein or lipids, mainly promote reactive oxygen species generation and a proinflammatory response through RAGE activation. Besides the cell membrane form, RAGE also exists as a soluble circulating molecule, sRAGE. This form, by binding the circulating AGEs and preventing their activation of RAGE, plays a role as an important protective agent [13, 14].

In renal diseases, AGEs and sRAGE may accumulate due to their increased formation and reduced elimination [15–19]. Indeed, the RAGE pathway has been suggested as a causal risk factor for both atherosclerosis [20] and left ventricular hypertrophy [21] in these patients.

Although the potential role of sRAGE as a marker for CVDs has been pointed out in different previous studies [22–27], its role in ESRD is less characterized.

To better evaluate the role of the AGEs/sRAGE pathway in ESRD, we firstly evaluated the existence of any potential association between AGEs, FGF-23, inflammation, and increased risk of CVDs in DM CKD-G5D patients. Secondarily, we explored the potential role of sRAGE as a marker of heart failure in CKD-G5D.

## 2. Materials and Methods

### 2.1. Source Population

We performed a cross-sectional study in patients on CKD-G5D. We enrolled patients who underwent hemodialysis (HD) or peritoneal dialysis (PD) treatment for at least 3 months with age ≥ 18 years and agreement to participate in the study. We excluded patients with missing or incomplete clinical history, incapacity to cooperate to the study, and hepatic encephalopathy. This study was performed in accordance with the ethical principles of the Declaration of Helsinki, as revised in 2013. The protocol was approved by the Ethics Committee of San Bortolo Hospital (N.41/14). All participants were informed of the objectives of the study and signed the informed consent.

### 2.2. Measurements

#### 2.2.1. Data Collection

Demographic, anthropometric, and clinical data (i.e., age, gender, smoking status, alcohol consumption, hypertension, DM, cardiovascular disease, and cerebrovascular disease) were collected. Screening and diagnosis of DM were performed according to the American Diabetes Association guidelines [28]. Hypertension was defined as values ≥ 140 mmHg systolic blood pressure and/or ≥90 mmHg diastolic blood pressure [29].

Blood samples in EDTA were collected during outpatient visits in PD patients or prior to dialysis treatment after long interdialytic intervals in HD patients. Samples for nonroutine assays were immediately frozen and stored at −80°C until measurements.

Concerning routine biochemical assays, total bilirubin (reference value (RV): male 0.3–1.5 mg/dL, female 0.2–1.2 mg/dL), calcium (RV: 8.5–10.5 mg/dL), phosphorous (RV: 2.2–4.2 mg/dL), LDL cholesterol (RV: <115 mg/dL), HDL cholesterol (RV: male > 40 mg/dL, female > 45 mg/dL), and total protein (RV: 6.4–8.7 g/dL) were quantified using colorimetric methods on Dimension Vista® 1500 Intelligent Lab System (Siemens, Milan, Italy). The same laboratory equipment was used for urea (RV: 15–50 mg/dL under 70 years old, 19–65 mg/dL over 70 years old), creatinine (RV: male up to 1.3 mg/dL, female up to 0.9 mg/dL under 70 years old and 1.2 mg/dL over 70 years old), uric acid (RV: male 3–8 mg/dL, female 2.4–6.6 mg/dL under 70 years old and 3–8 mg/dL over 70 years old), alanine aminotransferase (ALT) (RV: female < 31 U/L, male < 53 U/L under 70 years old and <34 U/L over 70 years old), and aspartate aminotransferase (AST) (RV: <37 U/L), which were all quantified by enzymatic methods, for total cholesterol (RV: <190 mg/dL) and triglycerides (RV: <150 mg/dL), both measured by kinetic enzyme assays, then for brain natriuretic peptide (BNP) (RV: <50 ng/L under 70 years old, <300 ng/L in the age range 51–75, and <600 ng/L over 70 years old) and C-reactive protein (CRP) (RV: <0.5 mg/dL), which were quantified by an immunochemiluminescent and a turbidimetric method, respectively. Sodium (RV: 35–145 mmol/L), potassium (3.3–5.0 mmol/L), and chloride (95–110 mmol/L) were measured on Dimension Vista System using ion-selective electrodes. Glucose (RV: <100 ng/mL) and albumin (RV: 2.1–4.5 g/dL) were quantified on the ILab650 system (Instrumentation Laboratory, A Werfen Company, Milan, Italy) using an enzymatic and a colorimetric method, respectively. Parathyroid hormone (intact PTH) (RV: 5–35 ng/L), 25-hydroxy vitamin D (25-(OH)D3) (RV: 30–100 *μ*g/L), and *β*2-microglobulin (*β*2-microglobulin: 0.8–2.5 mg/L) were measured using the Liaison XL system (DiaSorin, Vercelli, Italy) by immunochemiluminescent methods. The acid-base equilibrium (pH, HCO_3_^−^) (RV: 7.32–7.42 for pH, 22–29 mmol/L for HCO_3_^−^) was quantified by the Rapidpoint 405 Blood Gas Analyzer (Siemens).

#### 2.2.2. FGF-23 Quantification

The carboxyl-terminal (C-terminal) portion of FGF-23 (cFGF-23) levels was determined in plasma by two-site enzyme-linked immunosorbent assay (ELISA), according to the manufacturer's protocol (Immutopics Inc., San Clemente, CA). Two hundred microliters of plasma was used to assay the sample in duplicate. Samples with values greater than the highest standard were diluted 1 : 10 or greater with the 0 RU/mL standard or optional sample diluent reagent and reassayed. The lowest concentration of cFGF-23 measurable is 1.5 RU/mL, and the maximum intra- and interassay coefficients of variations were 2.4% and 4.7%, respectively.

#### 2.2.3. Glycated Albumin Quantification

The glycated albumin (GA) and the percentage of glycated albumin (GA%) were determined in plasma by the enzymatic QuantiLab® glycated albumin assay (Instrumentation Laboratory) using the ILab650 system (Instrumentation Laboratory). The ILab analyzer automatically calculates the results of each sample. The GA% is calculated by the GA/albumin ratio and corrected by arithmetic algorithm defined to align the GA% levels to the HPLC method [30–32]. The minimum detectable concentration of GA measurable is 1.15 g/L. The maximum intra- and interassay coefficient of variations were 2.1% and 1.3% for GA and 1.2% and 1.0% for GA%, respectively.

#### 2.2.4. sRAGE and Inflammatory Cytokine Quantification

The quantitative determinations of sRAGE, pentraxin-3 (PTX3), and tumor necrosis factor alpha (TNF*α*) concentrations were performed by commercial human ELISA kits (R&D System, Minneapolis, MN, USA) according to the manufacturer's instructions. The minimum detectable dose ranged from 1.23 to 16.14 pg/mL for sRAGE, 0.007 to 0.116 ng/mL for PTX3, and 0.5 to 5.5 pg/mL for TNF*α*. The maximum intra- and interassay coefficients of variations were, respectively, 4.8% and 8.3% for sRAGE, 4.4% and 6.2% for PTX3, and 5.2% and 7.4% for TNF*α*. The GloMax®-Multi Microplate Multimode Reader was used for photometric measurements (Promega, Milan, Italy).

### 2.3. Statistical Analysis

Qualitative variables are summarized as numbers and percentages; quantitative variables are expressed as mean with standard deviation (SD) or median and interquartile range (IQR). The normality of data distribution was assessed by the Kolmogorov-Smirnoff test. *t*-test and Mann–Whitney *U* test were used for group comparison. To test the univariate association between variables, Pearson (for normal-distributed data) or Spearman (for non-normal distributed data) correlation tests were used, as appropriate. Stepwise regression analysis was performed to evaluate the independent correlates of BNP in CKD-G5D patients. All statistical analyses were performed using STATISTIX 7.0 (Analytical Software, Tallahassee, FL, USA) and GraphPAd Prism 5.0 biochemical statistical package (GraphPad Software, San Diego, CA, USA). A *p* value < 0.05 was considered significant.

## 3. Results

### 3.1. Patient Characteristics

We enrolled a total of 76 CKD-G5D patients (32 HD, 44 PD, median age 62.41 (IQR: 52.02–72.05) years, 55M) of which 24 were with DM (type 2 DM: 22; type 1 DM: 2) (mean age 61.01 (50.94–72.83) years, 35M) and 54 were without DM (65.42 (54.83–70.94) years, 20M). Demographic and anthropometrical data are presented in Table 1. Sixty-seven patients (87%) were under treatment with vitamin D or its synthetic analog. The active vitamin D therapy, which included cholecalciferol and calcitriol, was used in 45 (59.21%) patients. Twenty-two (28.95%) patients were treated with paricalcitol and cinacalcet.

Biochemical characteristics of patients included in the study are shown in Table 2.

### 3.2. Plasma Levels of GA, FGF-23, sRAGE, and Inflammatory Markers

CKD-G5D patients were classified according to the presence of DM, and the two groups were compared to explore potential differences in the levels of GA, as a marker of protein glycation, sRAGE, cFGF-23, and the proinflammatory molecules CRP, PTX-3, and TNF*α*.

**Figure 1:**
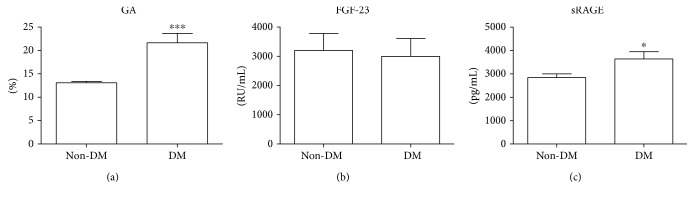
Evaluation of GA, FGF-23, and sRAGE levels in CKD-G5D patients. GA levels (a) and sRAGE (c) were higher in DM CKD-G5D patients than in non-DM CKD-G5D patients (^∗∗∗^*p* < 0.001 and ^∗^*p* < 0.05, resp.). FGF-23 levels (b) were the same in the two groups.

According to the reference limits of GA [33], which have been very recently documented also in Caucasians [34, 35] (upper reference limit: 14.5% (95% CI: 14.3–14.7) [34]; range: 9.0% (90% CI: 8.7–9.5) to 16.0% (90% CI: 15.6–16.4) [35]), in non-DM CKD-G5D patients, GA (95% CI: 12.52–13.66) was within the ranges of normality (Table 1 and Figure 1()). Differently, in the DM CKD-G5D group, it reached pathological levels and it was statistically significantly higher than in the non-DM CKD-G5D patients (*p* < 0.001) (Table 1 and Figure 1()). According to our previous results on sRAGE concentrations in healthy subjects (mean value 1363.0 ± 693.2 ng/mL) [36], sRAGE levels were above the normal values both in non-DM CKD-G5D and DM CKD-G5D patients and resulted statistically significantly higher in the DM CKD-G5D compared to the non-DM CKD-G5D group (*p* < 0.05) (Table 1 and Figure 1()). cFGF-23 levels were higher than the reference value (<180 RU/mL), but we did not find any significant difference between groups (non-DM CKD-G5D: median value, 1345.00, 25th–75th percentiles (508.10–3087.00) RU/mL; DM CKD-G5D: 1707.00, (1183.00–4016.00 RU/mL) (Table 2 and Figure 1())).

**Figure 2:**
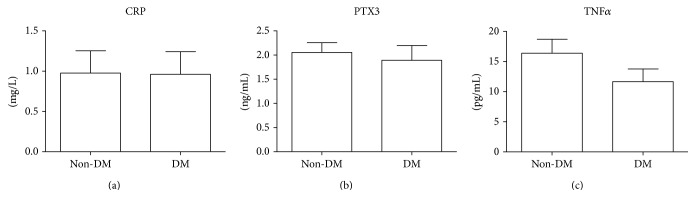
Evaluation of inflammation-related molecules in CKD-G5D patients. CKD-G5D patients were classified into two groups according to the presence of diabetes mellitus (DM). CRP (a), PTX3 (b), and TNF*α* (c) levels were compared between DM and non-DM groups. No statistically significant differences were observed between the two groups.

As markers of inflammation, we evaluated CRP, PTX-3, and TNF*α*. According to the existing reference values for healthy subjects (<0.5 mg/L for CRP, <1.18 ng/mL for PTX-3, and 1.12 pg/mL for TNF*α*, resp.), all the proinflammatory markers evaluated were greatly upregulated in both groups but without significant differences between them (Table 2 and Figure 2()).

A univariate association analysis was then performed in CKD-G5D patients to explore potential correlations between the markers previously studied. We did not find any significant correlation between GA and cFGF-23 (*r* = 0.073, *p* = 0.529), between sRAGE and cFGF-23 (*r* = −0.056; *p* = 0.633), and then between GA and sRAGE (*r* = 0.29, *p* = 0.061).

### 3.3. Relationships between CVD Risk Factors and BNP

The potential correlations between BNP, a marker used for screening and prognosis of heart failure, and clinical variables in CKD-G5D patients were explored. BNP was significantly positively correlated with creatinine (*r* = 0.27; *p* = 0.017), potassium (*r* = 0.247; *p* = 0.031), CRP (*r* = 0.260; *p* = 0.023), sRAGE (*r* = 0.314; *p* = 0.006), and *β*2-microglobulin (*r* = 0.407; *p* < 0.001) and significantly negatively correlated with sodium (*r* = −0.341; *p* = 0.003).

In a multivariate stepwise regression model, plasma sRAGE and CRP levels were the only independent predictors of BNP (Table 3). All the other parameters did not enter the model.

## 4. Discussion

CKD-G5D patients are an interesting model of premature aging. These patients, due to the lack of renal function, show a uremic milieu in which phosphate retention and uremic toxin accumulation, including AGEs, promote oxidative stress and inflammation. These conditions may in turn activate specific cellular mechanisms, such as telomere attrition, DNA damage, and mitochondrial dysfunction, which affect cellular homeostasis, promote premature cellular senescence, and increase the risk of death mainly due to cerebrovascular and cardiovascular complications [37].

AGEs are recognized as important damaging molecules for the cardiovascular system due to their ability to promote endothelial dysfunction, arterial stiffness, atherosclerosis, immune system alteration, and cardiac fibrosis and remodeling [38–41]. It is known that the generation of AGEs is strongly increased in DM, being AGEs by-products of hyperglycemia. Recent preclinical studies [11, 12] suggested that these molecules, in addition to their known role as proinflammatory agents, are able to increase the production of FGF-23, a key molecule involved in the crosstalk between kidney function, bone metabolism, and the cardiovascular system [7, 8, 42]. To our knowledge, this is the first study investigating in human any potential association between AGEs, sRAGE, cFGF-23, and cardiovascular complications in CKD-G5D patients with DM. Our results indicated that both GA and sRAGE levels were increased in DM CKD-G5D compared to non-DM CKD-G5D patients, but the levels of cFGF-23 did not differ between the two groups. Similarly, the concentrations of the proinflammatory molecules evaluated were almost the same in the two groups, although we expected to observe higher levels in DM CKD-G5D patients, as a consequence of the increased glycated milieu. To our opinion, one possible explanation just deals with the upregulation of sRAGE. Different studies have shown that sRAGE levels are increased in DM as a counteract system against glycated products [26, 43–46]. Assuming the activation of the same mechanism also in our DM CKD-G5D patients, sRAGE, by blocking glycated products, could reduce the activation of various damaging cellular mechanisms, including the stimulation of cFGF-23 and other proinflammatory molecules. Indeed, since AGE accumulation has been associated with the development and progression of heart failure [47, 48], the lack of difference also in BNP levels between the two groups reinforces the idea of a protective role of sRAGE in DM CKD-G5D patients. A further explanation could arise by considering that cFGF-23, which starts to rise early in CKD, in CKD-G5D is up to thousandfold higher than the normal levels [49]. For this reason, we could not exclude the possibility that in DM CKD-G5D patients, a further stimulation of the FGF-23 system by potential activators, like AGEs [11, 12], is not possible or may not be appreciated.

Concerning AGEs, we focused our attention on GA. As for other AGEs, we expected to observe that GA levels were over the ranges of normality not only in DM CKD-G5D group but also in non-DM CKD-G5D patients, due to the increased oxidative stress and the reduced kidney clearance typical of the disease [15, 17, 18, 50]. Of course, the upregulation of sRAGE at levels above controls in both groups and its further increase in DM seem to suggest the existence of a glycated milieu in all CKD-G5D patients, regardless of the presence of DM. According to these data, the observation that GA levels were over the range of normality only in DM CKD-G5D group strongly reinforces the utility of GA as a useful glycation marker for DM monitoring in CKD-G5D patients in which HbA1c does not work well due to just kidney-related anemia [51, 52].

sRAGE has been regarded as a diagnostic and prognostic marker of cardiovascular outcome in various pathological conditions, that is, obesity, DM, metabolic syndrome, chronic heart failure, and also CKD [20–22, 24, 26]. Concerning heart failure, conflicting results on the relationship between sRAGE and heart failure risk exist. Both lower and higher circulating levels of sRAGE were described as valuable predictors of heart failure, its severity, and mortality, and some studies suggested the existence of a robust association between NT-pro BNP levels, as a diagnostic and prognostic marker of heart failure and sRAGE [27, 48, 53–56]. Also, in our study, we observed a positive correlation between sRAGE and BNP. Indeed, sRAGE emerged as an independent predictor of BNP levels, thus suggesting its potential role as a marker of cardiac remodeling in CKD-G5D patients.

Leonardis et al. [21] studied the relationships between sRAGE and left ventricular hypertrophy in CKD, not in CKD-G5D. They showed that sRAGE levels were increased compared to those of controls but, unlike us, were inversely correlated with functional parameters of cardiac function. Probably, since the two studies have been performed on different populations, they are not easily comparable and further studies in ESRD are therefore necessary to support data herein presented.

The study of Kim et al. [20] has been performed on PD patients but explored sRAGE correlation with carotid atherosclerosis, not parameters of heart failure. Although different in its aim, some data of this study could be useful for a better comprehension also of our results. They observed that CKD-G5D patients had increased sRAGE levels compared to controls but, differently from our results, the subgroup of DM patients had lower sRAGE and higher IL-6 levels, a marker of inflammation, than the non-DM group. To our opinion, this observation seems to reinforce our hypothesis of a protective role of sRAGE against a further increase of the inflammatory status in DM patients. Anyway, the reasons of the different results are not clear but could deal with a different regulation of sRAGE expression at cellular level, the duration of disease, and features of patients included in the study.

In conclusion, in DM CKD-G5D patients, sRAGE appeared to be a marker of cardiac remodeling. Indeed, its increase could be a potential protective mechanism against the increased risk of cardiovascular complications related to AGEs and inflammation. The causal relationship between sRAGE and cardiovascular risk in these patients needs to be further confirmed by mechanistic studies. Also, the evaluation of additional glycated products, the quantification of sRAGE, secreted form of the receptor, and a comparison between HD and PD could help to improve the knowledge of the role of glycated pathways in these patients.

## Figures and Tables

**Table 1:** Demographic, anthropometric, and clinical characteristics of CKD patients included in the study.

	All CKD (*n* = 76)	Non-DM CKD (*n* = 52)	DM CKD (*n* = 24)	*p*
Age (years)	62.41 (52.02–72.05)	61.21 ± 13.94	65.42 (54.83–70.94)	0.80
Male gender (*n*, %)	55, 72.37%	35, 67.31%	20, 83.33%	0.18
BMI	27.20 ± 5.56	27.38 ± 5.28	24.14 (22.10–31.35)	0.50

HD (*n*, %)	32, 42.11%	20, 38.46%	12, 50%	0.45

Smoking (*n*, %)	9, 11.84%	4, 7.69%	5, 20.83%	0.13
Ex-smoking (*n*, %)	29, 38.16%	20, 38.46%	9, 37.50%	1.00
Alcohol consumption (*n*, %)	2, 2.63%	2, 3.85%	0, 0%	1.00
Hypertension (*n*, %)	48, 63.16%	35, 67.30%	13, 54.17%	0.31
Cardiovascular diseases (*n*, %)	22, 28.95%	12, 23.08%	10, 41.67%	0.11
Cerebrovascular diseases (*n*, %)	4, 5.26%	2, 3.85%	2, 8.33%	0.59

Therapy with activated vitamin D	45, 59.21%	31, 59.62%	14, 58.33%	1.00
Therapy with paricalcitol	22, 28.95%	17, 32.69%	5, 20.83%	0.05

Data are expressed as median (25th–75th percentiles) or number and proportions. BMI: body mass index; HD: hemodialysis. Comparison between groups was performed by Mann–Whitney *U* test or Fisher exact test.

**Table 2:** Biochemical characteristics of CKD-G5D patients included in the study.

	All CKD-G5D (*n* = 76)	Non-DM CKD-G5D (*n* = 52)	DM CKD-G5D (*n* = 24)	*p*
Creatinine (mg/dL)	9.40 ± 3.09	9.71 ± 3.39	8.71 ± 2.19	0.13
Uric acid (mg/dL)	5.70 ± 1.29	5.67 ± 1.24	5.76 ± 1.41	0.78
Urea (mg/dL)	124.60 ± 31.41	122.10 ± 31.60	129.90 ± 30.97	0.32
Total bilirubin (g/dL)	0.40 (0–30-0.50)	0.40 (0.30–0.50)	0.42 ± 0.16	0.56

pH venous	7.35 (7.32–7.38)	7.35 (7.32–7.38)	7.36 (7.33–7.38)	0.47
HCO_3_ venous (mmol/L)	25.18 ± 4.29	25.02 ± 4.54	25.53 ± 3.76	0.63
K—potassium (mmol/L)	4.46 ± 0.72	4.46 ± 0.71	4.48 ± 0.75	0.91
Na—sodium (mmol/L)	140.00 (138.00–142.00)	140.00 (138.30–142.80)	139.50 ± 3.02	0.33
Cl—chloride (mmol/L)	101.50 (97.00–105.00)	101.10 ± 4.28	101.20 ± 5.74	0.95
Ca—calcium (mg/dL)	8.95 ± 0.47	8.98 ± 0.48	8.88 ± 0.44	0.38
P—phosphorus (mg/dL)	5.15 ± 1.36	5.14 ± 1.34	5.20 ± 1.44	0.86

Total cholesterol (mg/dL)	157.10 ± 40.66	163.90 ± 35.42	142.50 ± 47.76	**0.032**
LDL cholesterol (mg/dL)	80.82 ± 35.92	85.65 ± 32.32	70.33 ± 43.17	**0.016**
HDL cholesterol (mg/dL)	44.00 (38.00–54.00)	45.50 (40.00–60.25)	42.42 ± 11.81	**0.030**
Triglycerides (mg/dL)	131.50 (86.25–201.80)	117.50 (82.75–204.30)	154.00 ± 74.15	0.55
ALT (UI/L)	19.00 (15.00–24.00)	20.12 ± 7.79	20.00 (15.25–31.25)	0.30
AST (UI/L)	11.00 (6.00–15.75)	11.00 (6.00–14.75)	11.42 ± 6.79	0.93
Glucose (mg/dL)	103.50 (92.00–138.50)	99.00 (91.00–110.00)	165.00 (131.80–220.80)	**<0.0001**
Total protein (g/dL)	7.00 ± 0.57	7.02 ± 0.63	6.96 ± 0.41	0.64
Albumin (g/dL)	35.03 ± 4.82	35.01 ± 4.81	34.70 (31.35–39.23)	0.75
GA%	14.00 (12.03–17.15)	13.09 ± 2.05	18.30 (17.13–23.20)	**<0.0001**

iPTH (pg/mL)	103.50 (53.25–211.00)	102.00 (55.00–254.30)	134.30 ± 119.90	0.46
25-(OH)D3 (ng/mL)	17.90 (10.93–23.28)	17.95 (11.45–25.15)	16.32 ± 6.77	0.30

CRP (mg/L)	0.34 (0.29–0.78)	0.31 (0.29–0.77)	0.41 (0.29–1.04)	0.39
*β*2-microglobulin (ng/mL)	22.80 (16.66–28.98)	22.96 (16.88–29.61)	21.08 ± 7.66	0.24
BNP (pg/mL)	2542.00 (1511.00–10762.00)	2265.00 (1108.00–8272.00)	3064.00 (1795.00–18558.00)	0.148

cFGF-23 (RU/mL)	1441.00 (759.00–3614.00)	1345.00 (508.10–3087.00)	1707.00 (1183.00–4016.00)	0.142
sRAGE (pg/mL)	3089.30 ± 1339.74	2838.00 ± 1163.75	3633.80 ± 1548.45	**0.015**
PTX3 (ng/mL)	1.57 (0.76–2.94)	1.57 (0.75–3.23)	1.89 ± 1.48	0.65
TNF*α* (pg/mL)	10.71 (4.85–21.34)	12.15 (5.60–24.19)	11.66 ± 10.28	0.134

Data are expressed as mean ± SD or median (25th–75th percentiles). ALT: alanine transaminase; AST: aspartate transaminase; BNP: brain natriuretic peptide; CRP: C-reactive protein; GA: glycated albumin; cFGF-23: c-terminal portion of fibroblast growth factor-23; iPTH: intact parathyroid hormone; 25-(OH)D3: 25-hydroxy vitamin D; PTX3: pentraxin-related protein PTX3; sRAGE: soluble receptor for advanced glycation end products; TNF*α*: tumor necrosis factor alpha. Comparison between groups was performed by unpaired *t*-test or Mann–Whitney *U* test. *p* values less than 0.05 are indicated in bold.

**Table 3:** Stepwise regression analysis (*t* value) of the association between some independent variables and BNP in CKD-G5D patients.

BNP (pg/mL)	Independent variables					Model *R*^2^
	CRP (mg/L)	Creatinin (mg/dL)	Na (mmol/L)	K (mmol/L)	sRAGE (pg/mL)	
	2.44	0.84	−0.81	1.40	2.72	0.20
*p* value	**0.017**	0.40	0.42	0.17	**0.008**	
Constant value						
Regression coefficient	1652.06				2.49	
SE regression coefficient	676.00				0.92	

CRP: C-reactive protein; sRAGE: soluble receptor of advanced glycation end products; SE: standard error.
